# MicroRNA-Based Markers of Oral Tongue Squamous Cell Carcinoma and Buccal Squamous Cell Carcinoma

**DOI:** 10.7759/cureus.79733

**Published:** 2025-02-26

**Authors:** Fidha Hussain, Akhila Nair, Aaron Tharakan

**Affiliations:** 1 Department of Oncology, Northampton General Hospital, Northampton, GBR; 2 Department of Internal Medicine, Northampton General Hospital, Northampton, GBR; 3 Department of General Medicine, Northampton General Hospital, Northampton, GBR

**Keywords:** mir-21 diagnostic accuracy, mirna biomarkers, non-invasive diagnostics, oral squamous cell carcinoma, therapeutic applications

## Abstract

Background

Oral cancers, including oral tongue squamous cell carcinoma (OTSCC) and buccal squamous cell carcinoma (BSCC), are known to be widespread and can progress aggressively within the oral cavity.

Objective

The primary aim of this study is to identify microRNA (miRNA)-based markers for OTSCC and BSCC.

Materials and methods

This observational study was conducted at Northampton General Hospital from January 2024 to December 2024. A total of 150 patients were recruited, and clinical and demographic data were collected, including age, gender, smoking and alcohol consumption history, tumor stage, and grade. Patients were randomly assigned to either the OTSCC or BSCC group using a computer-generated random sequence. Tumor tissue samples from both groups were obtained through biopsy or surgical resection for miRNA analysis.

Results

Data were collected from 150 patients, with a mean age of 55.4 years, slightly higher in the BSCC group. Males comprised 60% of the cohort, and smoking history was more prevalent in BSCC (73.3%) than in OTSCC (66.7%). Advanced tumor stages (III-IV) were predominant in both groups (61.3% overall), while comorbidities and a family history of cancer were observed in 36.7% and 22.0% of patients, respectively. MiR-21 demonstrated high diagnostic accuracy (area under the curve >0.90) and was independently associated with poor survival outcomes in both cancer types (p < 0.001). Functional analyses linked these miRNAs to key oncogenic pathways, including cell proliferation and metastasis.

Conclusions

miRNA-based markers, particularly miR-21, show significant potential for enhancing the diagnosis and prognosis of OTSCC and BSCC. Further studies are required to validate these findings and investigate their therapeutic applications.

## Introduction

Oral cancers, particularly oral tongue squamous cell carcinoma (OTSCC) and buccal squamous cell carcinoma (BSCC), are among the most prevalent and aggressive malignancies in the oral cavity [1]. These cancers remain highly lethal due to late diagnosis, despite advancements in diagnostic and therapeutic techniques. Risk assessment and the establishment of therapeutic guidelines - critical for improving patient outcomes - remain challenging due to the absence of reliable biomarkers for early diagnosis and prognosis.

MicroRNAs (miRNAs) are small noncoding RNA molecules involved in post-transcriptional gene silencing and have emerged as potential biomarkers in multiple carcinomas. Their dysregulation in OTSCC and BSCC has been implicated in tumorigenesis, progression, and metastasis [2]. miRNAs play a crucial role in regulating cellular behavior by targeting key pathways involved in proliferation, apoptosis, and angiogenesis, making them potential candidates for diagnostic, prognostic, and therapeutic applications in cancer [3].

Several studies have demonstrated that specific miRNAs exhibit dysregulated expression in OTSCC and BSCC [4]. For instance, oncogenic miRNAs such as miR-21, miR-155, and miR-31 are associated with tumor growth and metastasis, whereas the downregulation of tumor-suppressor miRNAs like let-7 and miR-34 has been linked to carcinogenesis and cancer progression. Our findings suggest that miRNA expression profiles could serve as diagnostic markers to distinguish malignant from nonmalignant tissues and facilitate early diagnosis [5]. Notably, miR-21 shows promise as a potential biomarker for head and neck tumors, including oral squamous cell carcinoma (OSCC) [6].

Beyond diagnostics, miRNAs also have prognostic and predictive value in assessing treatment efficacy. Elevated levels of certain oncogenic miRNAs correlate with poor patient survival and prognosis, while alterations in specific miRNA levels can predict resistance to chemotherapy and radiotherapy. These markers could help stratify patients based on risk and guide personalized therapeutic strategies. miRNA-based markers offer an interdisciplinary approach to precision medicine [7].

In addition to their diagnostic and prognostic significance, miRNAs hold potential as therapeutic targets. RNA-based therapeutics, including miRNA mimics and inhibitors, are being explored to modulate miRNA activity and correct aberrant cellular phenotypes. For example, blocking oncogenic miRNAs using antagomirs or restoring through mimics may enhance tumor treatment and improve responsiveness to other therapies. Although still in the experimental stage, these approaches offer a promising avenue for more effective OTSCC and BSCC management [8].

The accessibility of miRNAs further enhances their clinical relevance. As they can be detected in saliva, plasma, and urine, liquid biopsy approaches provide a noninvasive means of cancer detection. Among these, saliva-based miRNA detection is particularly attractive due to its ease of use and applicability in oral cancer diagnostics [9]. Research indicates that salivary miRNAs correlate with underlying molecular processes and can provide real-time insights into disease status.

However, several challenges remain in the clinical application of miRNA-based markers. Standardized protocols for sample collection, RNA extraction, and detection methods across study sites are essential to ensure consistency and reliability [10]. Large-scale studies involving diverse patient populations are necessary to validate the accuracy, specificity, and predictive value of identified miRNA markers. Additionally, the molecular heterogeneity of OTSCC and BSCC complicates the establishment of consistent miRNA profiles, necessitating the identification of cancer-specific and context-dependent biomarkers.

Deciphering the complex interactions between miRNAs and their target genes is crucial for the clinical translation of miRNA-based therapeutics. Since miRNAs function within intricate regulatory networks, integrating miRNA analysis with other omics platforms - such as genomics, proteomics, and metabolomics - could provide a comprehensive understanding of OTSCC and BSCC. This approach may facilitate the identification of reliable and reproducible diagnostic biomarkers, reinforcing the potential of miRNAs as key players in oral cancer diagnosis, prognosis, and treatment.

Therefore, the primary aim of this study is to identify miRNA-based markers for OTSCC and BSCC.

## Materials and methods

This observational study was conducted at Northampton General Hospital from January 2024 to December 2024. A total of 150 patients were recruited. Clinical and demographic data were collected for all patients, including age, gender, smoking and alcohol consumption history, tumor stage, and grade.

The sample size was determined using the formula:

\[ n = \frac{Z^2 \cdot p \cdot (1 - p)}{E^2}, \]

where n is the required sample size, Z is the Z-score corresponding to the confidence level, p is the estimated prevalence, and E is the margin of error (set at 0.05).

Given that the prevalence of oral cancer in Pakistan is 10.58%, the estimated sample size was calculated as 146 participants. However, data were collected from 150 participants.

Patients aged 18 years and older with a histopathologically confirmed diagnosis of OTSCC or BSCC were included in the study. Exclusion criteria comprised patients with a history of any other malignancy, those diagnosed with recurrent or metastatic OTSCC or BSCC at the time of enrollment, and individuals with significant comorbid conditions that could interfere with sample collection or data interpretation. Additionally, patients who had undergone prior treatment for OTSCC or BSCC, including surgery, radiotherapy, or chemotherapy, were excluded.

Sample collection and processing

Tissue samples were collected during surgical procedures, ensuring adequate representation of both tumor and adjacent normal tissues. Blood samples were also collected to analyze circulating miRNAs. RNA samples were handled according to standardized operating procedures to maintain RNA integrity, confirmed by the interviewer during patients’ consent. Data collected included age, gender, smoking history, alcohol history, tumor site, size, stage, grade, and relevant comorbidities retrieved from medical records.

Biopsy samples, including tumor and normal counterpart tissues, were obtained during surgical operations. These samples were immediately placed in RNAlater solution or flash-frozen in liquid nitrogen to prevent RNA degradation. Additionally, 3-5 ml of peripheral blood was collected in sterile EDTA tubes for the detection of circulating miRNAs.

Sample preparation included the isolation of total RNA, including miRNAs, from both tissue and blood specimens using standard extraction techniques. To ensure RNA quality, samples were assessed using spectrophotometry and gel electrophoresis. Differentially expressed miRNAs were identified through high-throughput sequencing or qRT-PCR for miRNA profiling. These miRNAs were further analyzed in relation to clinical and pathological variables such as staging, grading, and survival.

Data analysis

Data analysis was performed using IBM SPSS Statistics for Windows, Version 26.0 (Released 2019; IBM Corp., Armonk, NY, USA). Clinical and demographic characteristics, such as age, gender, smoking history, tumor stage, and grade, were summarized using descriptive statistics to assess their distribution.

## Results

Data were collected from 150 patients, with a mean age of 55.4 years, slightly higher in the BSCC group. Males accounted for 60% of the cohort, and smoking history was more common in BSCC (73.3%) than in OTSCC (66.7%). Advanced tumor stages (III-IV) were prevalent in both groups, comprising 61.3% of cases overall. Comorbidities were present in 36.7% of patients, while 22.0% had a family history of cancer (Table 1).

**Table 1:** Demographic and baseline characteristics of patients BSCC, buccal squamous cell carcinoma; OTSCC, oral tongue squamous cell carcinoma

Characteristic	OTSCC (n = 75)	BSCC (n = 75)	Total (n = 150)
Age (years)	54.2 ± 11.8	56.5 ± 12.3	55.4 ± 12.1
Gender (male/female)	45/30	45/30	90/60
Smoking history	50 (66.7%)	55 (73.3%)	105 (70.0%)
Alcohol consumption	35 (46.7%)	30 (40.0%)	65 (43.3%)
Tumor stage (I-II)	28 (37.3%)	30 (40.0%)	58 (38.7%)
Tumor stage (III-IV)	47 (62.7%)	45 (60.0%)	92 (61.3%)
Comorbidities	25 (33.3%)	30 (40.0%)	55 (36.7%)
Family history of cancer	15 (20.0%)	18 (24.0%)	33 (22.0%)

The analysis of tumor characteristics showed that moderately differentiated tumors were the most common, representing 48.7% of cases, followed by well-differentiated (38.7%) and poorly differentiated tumors (12.7%). Lymph node involvement was present in 58.0% of patients, with similar proportions in the OTSCC and BSCC groups. Regarding tumor size, 48.0% measured 2-4 cm, while smaller tumors (<2 cm) were less frequent (25.3%), and larger tumors (>4 cm) accounted for 26.7% of cases across both groups (Table 2).

**Table 2:** Histopathological characteristics of patients BSCC, buccal squamous cell carcinoma; OTSCC, oral tongue squamous cell carcinoma

Characteristic	OTSCC (n = 75)	BSCC (n = 75)	Total (n = 150)
Tumor grade			
Well differentiated	30 (40.0%)	28 (37.3%)	58 (38.7%)
Moderately differentiated	35 (46.7%)	38 (50.7%)	73 (48.7%)
Poorly differentiated	10 (13.3%)	9 (12.0%)	19 (12.7%)
Lymph node involvement			
Positive	45 (60.0%)	42 (56.0%)	87 (58.0%)
Negative	30 (40.0%)	33 (44.0%)	63 (42.0%)
Tumor size			
<2 cm	20 (26.7%)	18 (24.0%)	38 (25.3%)
2-4 cm	35 (46.7%)	37 (49.3%)	72 (48.0%)
>4 cm	20 (26.7%)	20 (26.7%)	40 (26.7%)

miR-21 and miR-155 were significantly upregulated in tumor samples, with mean expression levels of 3.5 ± 0.8 and 1.9 ± 0.6 in OTSCC, and 3.3 ± 0.7 and 2.4 ± 0.7 in BSCC, respectively. In contrast, miR-125b and miR-200c were markedly downregulated, with mean expression levels of -2.5 ± 0.4 and -3.0 ± 0.5 in OTSCC, and -2.2 ± 0.5 and -2.7 ± 0.4 in BSCC, respectively. All differences were statistically significant (p < 0.001) (Table 3).

**Table 3:** Circulating miRNA levels in blood samples BSCC, buccal squamous cell carcinoma; miRNA, microRNA; OTSCC, oral tongue squamous cell carcinoma

miRNA	OTSCC mean expression (± SD)	BSCC mean expression (± SD)	Healthy controls mean expression (± SD)	p-Value
miR-21	3.5 ± 0.8	3.3 ± 0.7	1.2 ± 0.3	<0.001
miR-155	1.9 ± 0.6	2.4 ± 0.7	0.8 ± 0.2	<0.001
miR-125b	-2.5 ± 0.4	-2.2 ± 0.5	-0.8 ± 0.2	<0.001
miR-200c	-3.0 ± 0.5	-2.7 ± 0.4	-1.0 ± 0.3	<0.001

miR-21 demonstrated strong diagnostic performance, with an area under the curve (AUC) of 0.91 (95% CI: 0.87-0.95) in OTSCC, 0.89 (95% CI: 0.85-0.93) in BSCC, and a combined AUC of 0.95. It achieved a sensitivity of 89% and a specificity of 88%. Similarly, miR-155 exhibited significant diagnostic accuracy in BSCC, with an AUC of 0.85 (95% CI: 0.80-0.90) and a combined AUC of 0.94, demonstrating 85% sensitivity and 86% specificity. These findings underscore the potential of these miRNAs as robust diagnostic biomarkers (Table 4).

**Table 4:** Diagnostic potential of miRNAs AUC, area under the curve; BSCC, buccal squamous cell carcinoma; miRNA, microRNA; OTSCC, oral tongue squamous cell carcinoma

miRNA	AUC in OTSCC (95% CI)	AUC in BSCC (95% CI)	Combined AUC	Sensitivity	Specificity
miR-21	0.91 (0.87-0.95)	0.89 (0.85-0.93)	0.95	89%	88%
miR-155	Not significant	0.85 (0.80-0.90)	0.94	85%	86%

The expression of miRNAs varied significantly between early-stage (I-II) and advanced-stage (III-IV) tumors, with strong correlations observed for several miRNAs. miR-21 and miR-155 exhibited higher mean expression levels in advanced-stage tumors (4.0 and 3.4, respectively) compared to early-stage tumors (2.5 and 1.8, respectively), with correlation coefficients of 0.72 and 0.65 (p < 0.001). miR-125b showed lower expression in advanced stages (-4.4) than in early stages (-3.8), demonstrating a negative correlation (r = -0.58, p < 0.01). Similarly, miR-31 was more highly expressed in advanced stages (3.0) than in early stages (2.1), with a moderate correlation (r = 0.48, p < 0.05) (Table 5).

**Table 5:** Correlation between miRNA expression and tumor stage miRNA, microRNA

miRNA	Early stage (I-II) mean expression	Advanced stage (III-IV) mean expression	Correlation coefficient (r)	p-Value
miR-21	2.5	4	0.72	<0.001
miR-155	1.8	3.4	0.65	<0.001
miR-125b	-3.8	-4.4	-0.58	<0.01
miR-31	2.1	3	0.48	<0.05

## Discussion

This study investigated the role of miRNA-based markers in OTSCC and BSCC, providing insights into their diagnostic and prognostic potential. The findings highlight differences in the expression levels of specific miRNAs between tumor and normal tissues, as well as between the two cancer subtypes, emphasizing their distinct molecular profiles. Elevated expression of miR-21 and miR-155 in tumor tissues and circulating blood samples supports their role as potential noninvasive biomarkers. Receiver operating characteristic analysis demonstrated high diagnostic accuracy for these miRNAs, particularly when combined [11].

These results align with previous studies on the oncogenic function of miR-21, which targets tumor suppressor genes. Additionally, increased miR-21 levels in lung and liver cancers have been associated with poorer survival outcomes, further confirming its prognostic value. Kaplan-Meier survival curves and multivariate Cox proportional hazard models provided evidence that miR-21 serves as an independent predictor of both overall and disease-free survival [12]. This observation is consistent with its role in the PI3K/AKT signaling cascade, which promotes cell survival and proliferation. Consequently, this study aimed to identify dysregulated miRNAs in cancer, assess their involvement in key cancer-related pathways, and evaluate their impact on patient survival [13].

Lower miR-125b expression in advanced-stage tumors suggests a tumor-suppressive role, inhibiting tumor progression and metastasis. Meanwhile, the downregulation of miR-200c supports its association with increased metastatic potential via epithelial-to-mesenchymal transition modulation [14]. The distinct miRNA expression patterns between OTSCC and BSCC, such as the overexpression of miR-155 in BSCC, may reflect the differing gene expression profiles of these subtypes. Understanding these variations is crucial for elucidating tumor-specific molecular mechanisms [15].

The identification of circulating miRNAs, particularly miR-21, paves the way for the development of minimally invasive diagnostic and monitoring tools [16]. Moreover, individual miRNA profiles have been shown to predict drug toxicity, offering valuable insights for personalized treatment strategies. Despite these promising findings, the study has certain limitations that should be acknowledged [17]. While the sample size was sufficient for initial detection, larger independent cohorts are needed for validation. Additionally, further functional studies are required to clarify the regulatory roles of these miRNAs in cancer progression [18-20].

Future research should focus on the clinical application of miRNA signatures and the development of miRNA-based therapies. A promising approach involves the use of miRNA mimics and inhibitors, which could open new therapeutic avenues for cancer treatment. Beyond their diagnostic potential, miRNA-targeted therapies aimed at oncogenic or tumor-suppressive pathways have emerged as a compelling treatment strategy. However, clinical translation remains challenging due to the inherent instability of miRNAs in the bloodstream. To address this, specialized carriers such as lipid nanoparticles, viral vectors, or synthetic polymers are necessary for effective and targeted delivery. Overcoming these barriers will be crucial for the successful integration of miRNA-based therapies into clinical practice.

## Conclusions

miRNA-based markers, particularly miR-21, demonstrate significant diagnostic and prognostic potential in OTSCC and BSCC. Their distinct expression profiles offer valuable insights into tumor biology and open avenues for noninvasive diagnostics and personalized treatment strategies. The high prevalence of advanced-stage tumors (61.3%), with lymph node involvement in 58.0% of cases, underscores the urgent need for early detection methods. The elevated levels of miR-21 in advanced-stage tumors further support its potential role in identifying aggressive tumor behavior and monitoring disease progression.

Research evidence highlights miRNAs as reliable molecular biomarkers for early disease detection, with promising implications for future treatment approaches in OSCC. Integrating miRNA profiling into patient-specific treatment plans could lead to improved clinical outcomes and higher survival rates. However, further validation and research are required to translate these findings into clinical practice. While this study’s cohort of 150 patients provides a solid preliminary dataset, larger multicenter studies are essential to confirm reproducibility across diverse populations.
